# Graphene Quantum Dot-Based Gold-Nickel Micromotors for Sensitive Detection of Ferric Ions

**DOI:** 10.1007/s10895-025-04238-6

**Published:** 2025-03-18

**Authors:** Gozde Yurdabak Karaca

**Affiliations:** Department of Medical Services and Techniques, Isparta Health Services Vocational School, Suleyman Demirel University, Isparta, 32260 Turkey

**Keywords:** Micromotor, GQD, Metal, Fe^3+^

## Abstract

The development of nano and micromotors has revolutionized the field of nanotechnology, offering innovative solutions for applications in biomedical engineering, environmental monitoring, and chemical sensing. Among these nano/micromotors, graphene quantum dot (GQD)-based micromotors have gained significant attention due to their unique optical and electronic properties. This study presents the synthesis and characterization of novel graphene quantum dot-based gold-nickel (GQD-Au-Ni) micromotors. These micromotors were synthesized using an electrochemical template deposition process, allowing precise control over their composition and structure. The GQD-Au-Ni micromotors exhibit multifunctionality, employing fluorometric, magnetic, and electrochemical methods for the selective and sensitive detection of ferric ions (Fe³⁺), with a remarkable limit of detection (LOD). The study highlights the potential of these micromotors in environmental monitoring paving the way for future research into multifunctional micromotors for a wide range of applications. The findings underscore the promise of GQD-based systems in advancing sensor technology and addressing critical challenges in environmental and health monitoring.

**Supplementary Information:**

The online version contains supplementary material available at 10.1007/s10895-025-04238-6.

## Introduction

The development of nano and micromotors has emerged as a groundbreaking advancement in the field of nanotechnology, offering promising applications across various domains, including biomedical engineering, environmental monitoring, and chemical sensing [1–3]. These tiny devices, capable of autonomous movement, have the potential to revolutionize the way we approach tasks at the micro and nanoscale, particularly in environments where traditional methods are ineffective or impractical [4–7]. Among the various types of nano/micromotors, those based on graphene quantum dots (GQDs) have garnered significant attention due to their unique optical and electronic properties [8, 9]. Graphene quantum dots are nanoscale fragments of graphene that exhibit quantum confinement and edge effects, leading to exceptional fluorescence characteristics [10]. These properties make GQDs ideal candidates for sensing applications, where sensitivity and selectivity are paramount [11–14]. As a promising nanomaterial, graphene quantum dots (GQDs) have opened a new area for sensor development, offering numerous benefits. GQDs display remarkable photoluminescence (PL) due to their specific functional groups, allowing them to interact with target analytes through various mechanisms such as electrostatic interactions, electron transfer, or π-π stacking [15]. These interactions can cause a turn-on or turn-off effect in the PL of GQDs. Additionally, this can lead to a reduction in the fluorescence intensity of GQDs. Graphene quantum dots (GQDs) have served as a fluorescent platform for the detection of a wide range of metal ions, biomolecules, and pesticides [15]. Sensors have been created to detect metal ions such as Fe³⁺, Cu²⁺, Zn²⁺, Cd²⁺, Ag²⁺, and Co²⁺. For example, rice husk-GQDs were prepared from rice husk as a precursor using the hydrothermal method and displayed the selectivity and sensitivity of Fe^3+^ ions compared to the other ions and showed a reasonable 5.8 nM limit of detection (LOD) value [16]. Another study showed the GQDs were functionalized with polyethyleneimine and observed as a photoluminescence sensor for sensing Fe^3+^ and Cu^2+^ ions [17]. Also, graphene-based nanomaterials hold significant promise for a variety of electrochemical biosensors because of their straightforward synthesis, excellent surface functionality, and high biocompatibility. The GQDs/graphene/glass carbon electrode demonstrated strong electrochemical responses using differential pulse anodic stripping voltammetry, attributed to the rapid electron transfer from graphene to GQDs, and exhibited a high affinity for Cu²⁺ [18]. Also, another study exhibited that nitrogen-doped graphene quantum dots can detect the concentration of Fe^3+^ in the Yellow River water [19].

This study was focused on the application of graphene quantum dot-based gold-nickel (GQD-Au-Ni) micromotors as fluorescence sensors for the detection of ferric ions (Fe³⁺). This is the first tubular micromotor GQD-Au-Ni in the literature. The detection of Fe³⁺ is of particular interest due to its critical role in various biological and environmental processes. Excessive levels of ferric ions can lead to severe environmental and health issues, necessitating the development of efficient and reliable detection methods [20, 21]. The GQD-Au-Ni micromotors were synthesized using an electrochemical template deposition process, which allows for precise control over the composition and structure of the micromotors [22, 23]. This method not only facilitates the integration of multiple functional materials but also enhances the micromotors’ performance in terms of speed and sensitivity. Our research demonstrates the multifunctionality of these micromotors, employing fluorometric, magnetic, and amperometric methods to achieve selective and sensitive detection of Fe³⁺ ions. The study provides a novel strategy for the development of advanced micromotors with potential applications in environmental monitoring and biomedical diagnostics.

In summary, this work presents a significant advancement in the field of micromotor technology, highlighting the potential of GQD-based systems in the sensitive detection of heavy metal ions. The findings of this study pave the way for future research into the development of multifunctional micromotors for a wide range of applications.

## Experimental

### Materials and Methods

Electrochemical deposition of micromotors was conducted using a CHI 720E potentiostat from CHInstruments. To measure and display the speed and fluorescence intensity of the micromotors, a Nikon Instrument Inc. Ti Optic LV100NDModel optical microscope was employed. Videos capturing the motion of the robots were recorded at a magnification of 40x with a frame rate of 5 frames per second. For fluorescence measurements, a DAPI filter (green filter, excitation at 470 nm) was utilized. The structural morphology and elemental composition of the GQD/Au/Ni micromotors were analyzed using scanning electron microscopy-energy dispersive X-ray spectroscopy (SEM-EDS) with an FEI Quanta FEG250 Model. Polycarbonate membranes with a diameter of 2 μm were sourced from Whatman, specifically the Cyclopore polycarbonate membranes (Catalog No 7060e2511, Whatman, Maidstone, UK). Nickel (II)sulfate hexahydrate (NiSO_4_·6H_2_O), Nickel (II) chloride hexahydrate (NiCl_2_·6H_2_O) and boric acid (H_3_BO_3_) were supplied by Sigma-Aldrich. Commercial gold plating solution was supplied from Orotemp 24 RTU RACK; Technic, Inc., Anaheim, CA, USA. Green Graphene Quantum Dots‚ Solution purchased from ACS Material (CAS NO: 7440-40-0).

The structural morphology and elemental analysis of the GQD-Au-Ni micromotor were examined using scanning electron microscopy- energy dispersive X-ray spectroscopy (SEM-EDS), FEI Quanta FEG 250 Model. UV − vis spectroscopy analysis was performed using a PerkinElmer UV/vis spectrometer, Lambda 20. Maxtek Inficon RQCM (RQCM Maxtek, USA.) Electrochemical characterization studies of micromotors were conducted using the CH Instruments 720E model potentiostat device and Metrohm DropSens DRP-DSC 7,000,029 screen-printed carbon electrode (SCPE) connector.

### Synthesis of GQD-Au-Ni Micromotors

A porous polycarbonate membrane served as the template. It was coated with a thin silver layer using RF magnetron sputtering. The sputtering parameters included a working pressure of 2 × 10^− 2^ mTorr, an RF power of 50 Watts, and a target-substrate distance of 6 cm. The silver coating achieved a thickness of approximately 80 nm using argon gas. The electrodeposition was conducted in a plating cell where the silver-coated polycarbonate membrane acted as the working electrode, with aluminum foil as the substrate. An Ag/AgCl reference electrode and a platinum wire counter electrode were employed. The deposition sequence for the GQD-Au-Ni micromotors involved applying the outer graphene quantum dot (GQD) layer at a potential of + 0.9 V for a charge of 1.5 C, followed by the gold (Au) layer at -1 V for 0.5 C, and finally the magnetic nickel (Ni) layer at -1.3 V for 10 C. After the plating process was finished, the conductive silver layer was meticulously etched using an alumina slurry with a particle size of 3–4 μm. To produce micromotors, the membrane was dissolved in dichloromethane (DCM) for 20 min, followed by a washing process. The washing involved centrifugation at 6000 rpm for 3 min, repeated three times with DCM, three times with ethanol (from Sigma Aldrich, 99.99% purity), and three times with distilled water. To ensure complete dissolution, each washing step included approximately 30 s of vortexing. Finally, the robots were stored in ultra-pure water at room temperature. Micromotors speeds were measured according to the applied magnetic field between 2 mT and 22 mT.

### Metal Incubation

**Scheme 1:**
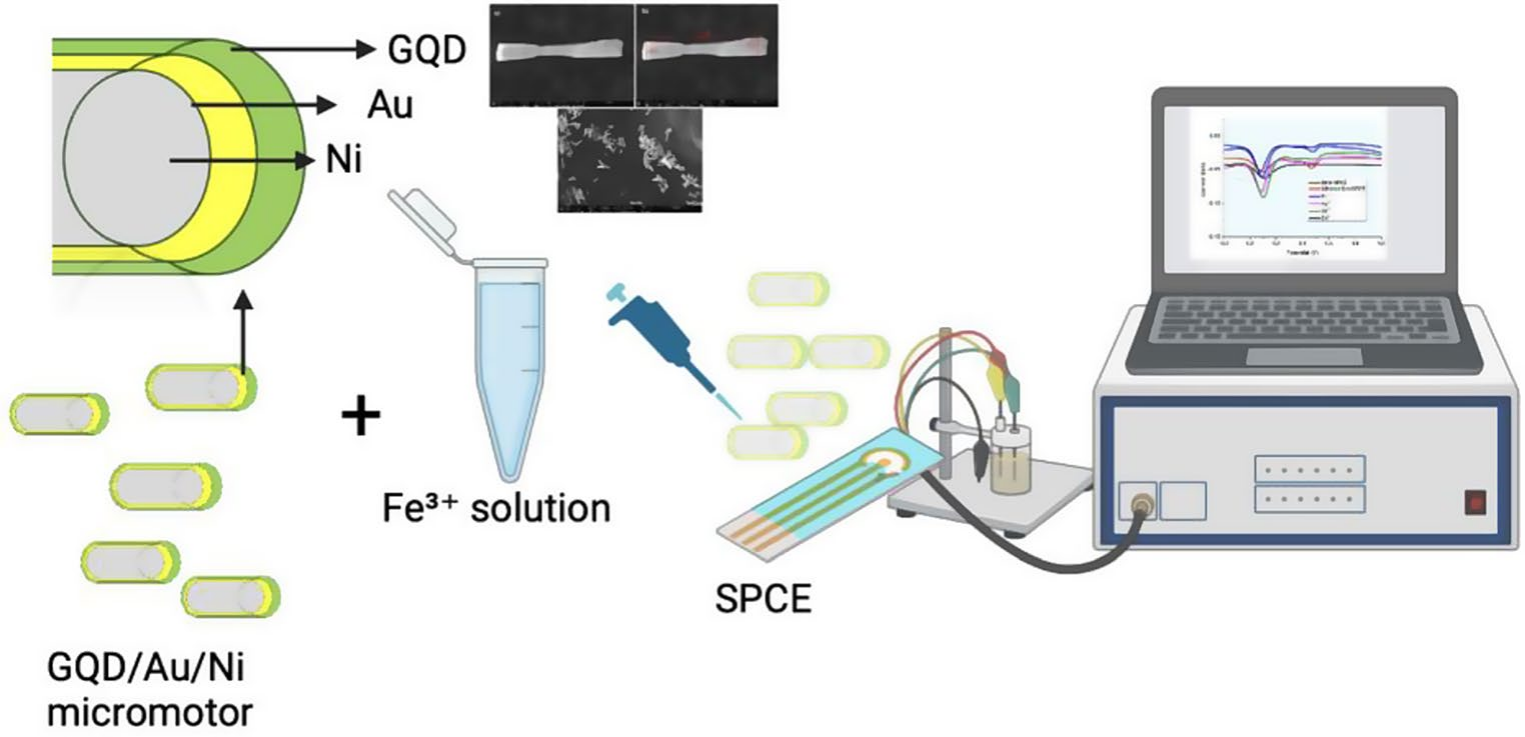
Experimental setup

The GQD-Au-Ni micromotors were incubated with various concentrations of ferric ions (Fe³⁺), ranging from 10^− 5^ M to 10^− 12^M, to observe changes in fluorescence intensity and speed. The limit of detection (LOD) for Fe³⁺ was determined using differential pulse voltammetry (DPV) and fluorescence intensity measurements. Additionally, the micromotors were tested for their selectivity by incubating them with other metal ions, including Zn, Sn, and Hg, to compare the fluorescence intensities and confirm their specificity for ferric ions. Electrochemical analysis was conducted using a screen-printed carbon electrode (SPCE) in a 0.1 M KCl solution containing 5 mM Fe(CN)₆³⁻/⁴⁻ as a redox probe to evaluate the surface characteristics of the micromotors. Scheme 1 shows the experimental setup for electrochemical detection of ferric ions.

## Results and Discussions

**Fig. 1:**
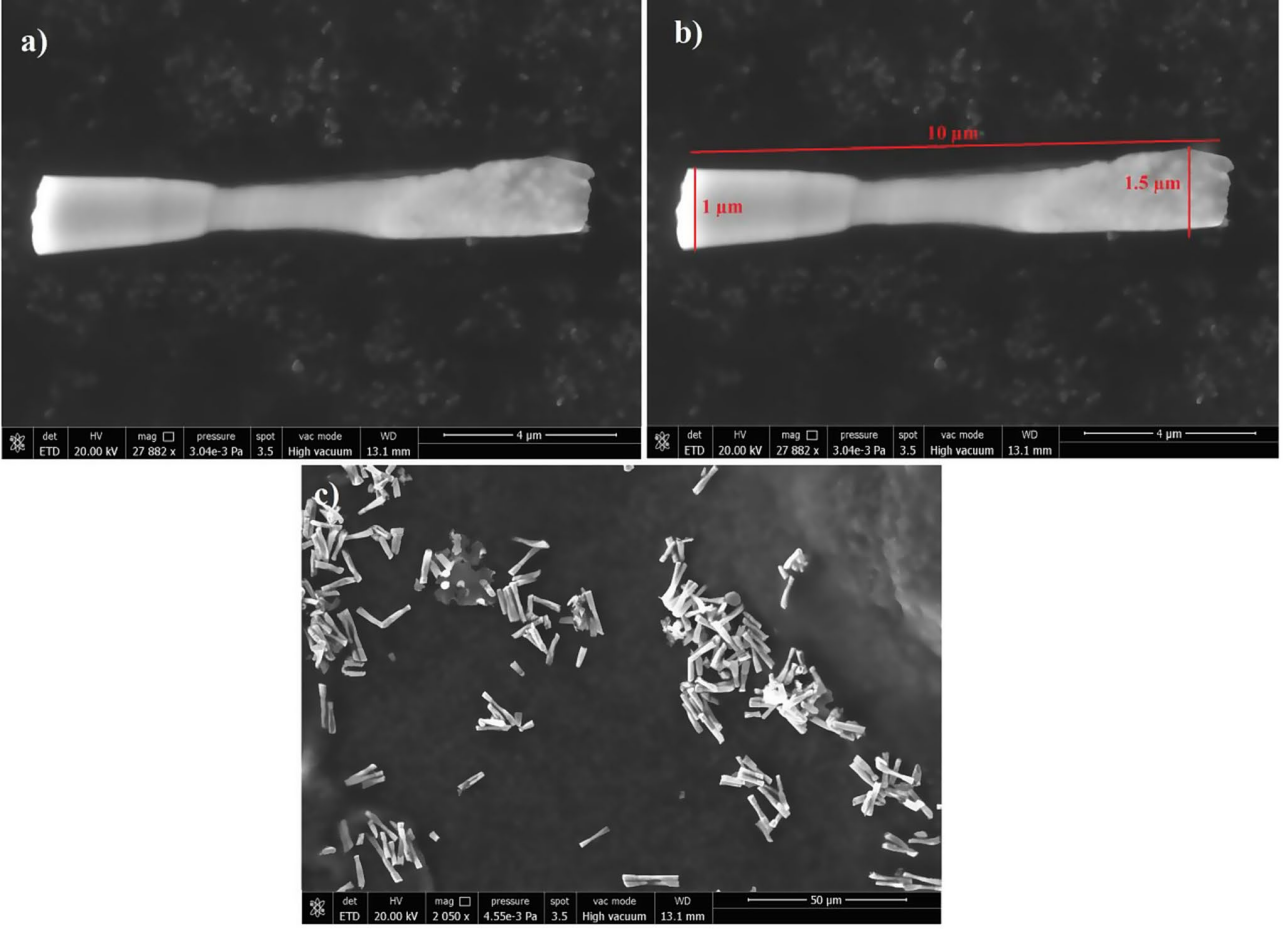
**a**) SEM image of GQD-Au-Ni micromotors **b**) SEM image includes measurements **c**) SEM images of micromotors at a lower magnification

Figure 1 shows the SEM images provided illustrate that the microstructural characteristics of GQD-Au-Ni micromotors. Figure 1a shows a single micromotor revealing its elongated structure with a smooth surface. Figure 1b presents the same micromotor with precise measurements, indicating a length of 10 μm and a width of 1.5 μm at the narrowest point. Figure 1c displays a collection of these micromotors at a higher magnification, showing a random distribution of microtubular structures. The mapping analysis of the GQD-Au-Ni micromotors, as supported by the provided data presented in SI Fig. 1, highlights the integration and distribution of materials within the micromotors. The analysis shows a significant presence of graphene quantum dots (GQD), gold (Au), and nickel (Ni). SI Fig. 1b shows all elements, showing a composite view where the predominant green (66%) indicates Carbon from graphene on the micromotor surface and base line from carbon tape. SI Fig. 1c highlights the blue component Oxygen. SI Fig. 1d focuses on the red Nickel element, suggesting a more localized distribution, representing a magnetic functionality. SI Fig. 1e emphasizes the yellow gold element.

**Fig. 2:**
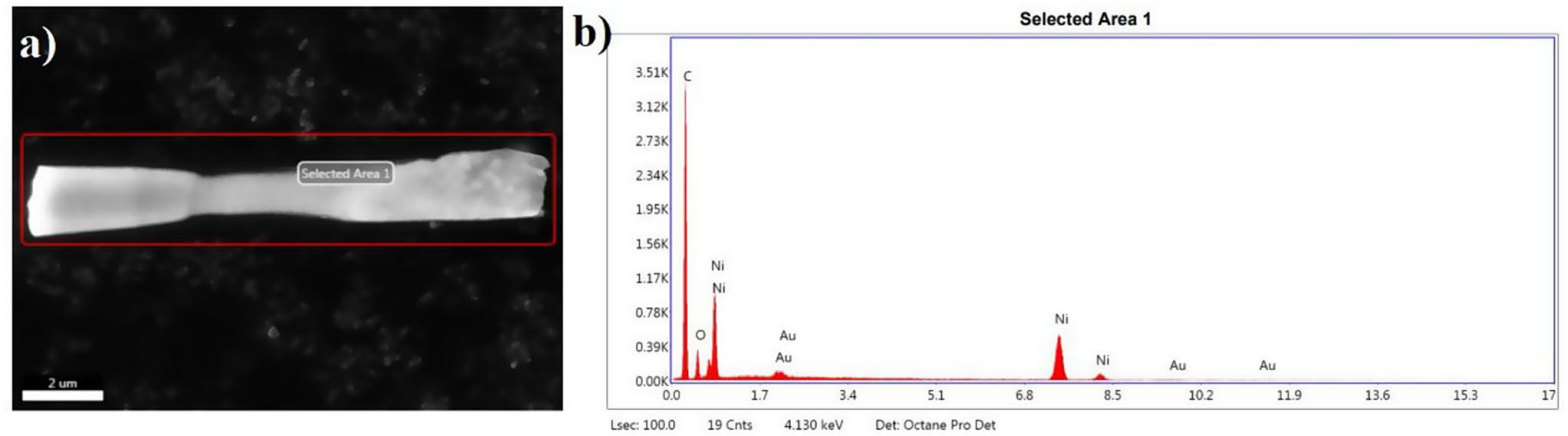
EDX results of GQD-Au-Ni micromotors (**a**) an SEM image highlighting the selected area for analysis, and (**b**) an EDX spectrum of that area

In the SEM image, the selected area is clearly marked, showing a uniform structure at a scale of 2 μm. The EDX spectrum in Fig. 2 reveals the elemental composition within this area, with prominent peaks for carbon (C), nickel (Ni), and gold (Au), indicating their significant presence. According to EDX results, 66.63% C, 10.69% O, 20.15 Ni, 2.53 Au was found as a weight%. These results are consistent with mapping results in SI Fig. 1.

**Fig. 3:**
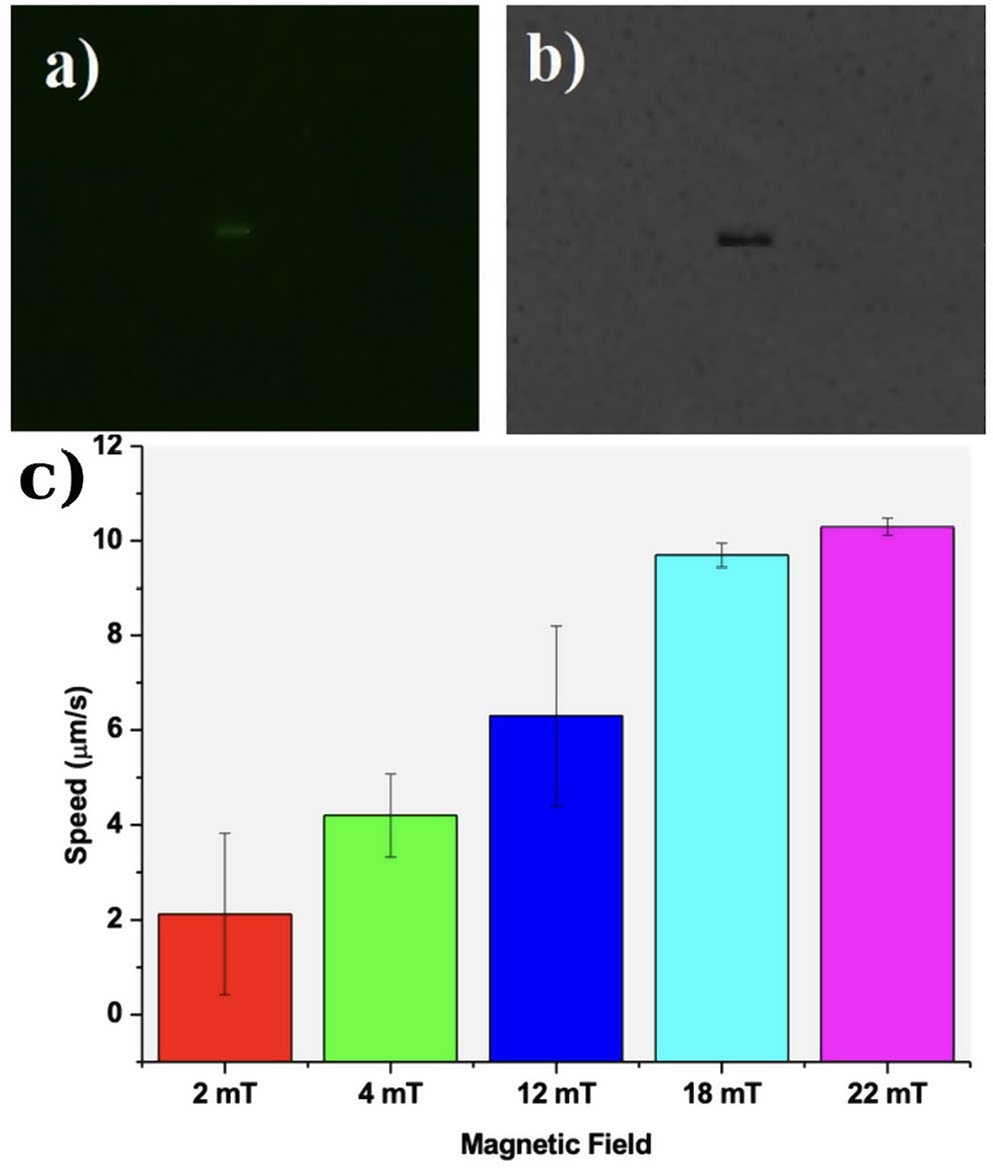
**a**) Fluorescence microscopy image (40× magnification, green filter, excitation at 420 nm) **b**) Optical microscopy image at 40× magnification **c**) Micromotor speed under different magnetic fields

Figure 3 shows the magnetic GQD-Au-Ni micromotor optical microscopy analysis results. Figure 3a shows fluorescence microscopy image of GQD-Au-Ni micromotor. Fluorescence intensity measured as 4050 a.u. at 40x magnification and 420 nm excitation. Figure 3b shows the optical microscopy image of GQD-Au-Ni micromotor. Figure 3c shows a bar graph illustrating the relationship between magnetic field strength (in mT) and speed (in µm/s). The graph shows that as the magnetic field increases from 2 mT to 22 mT, the speed of the micromotor also increases 2 to 10.3 μm/s [22]. Also, SI Video 1 shows the movement of GQD- Au-Ni micromotor movement under 22 mT magnetic field.

**Fig. 4:**
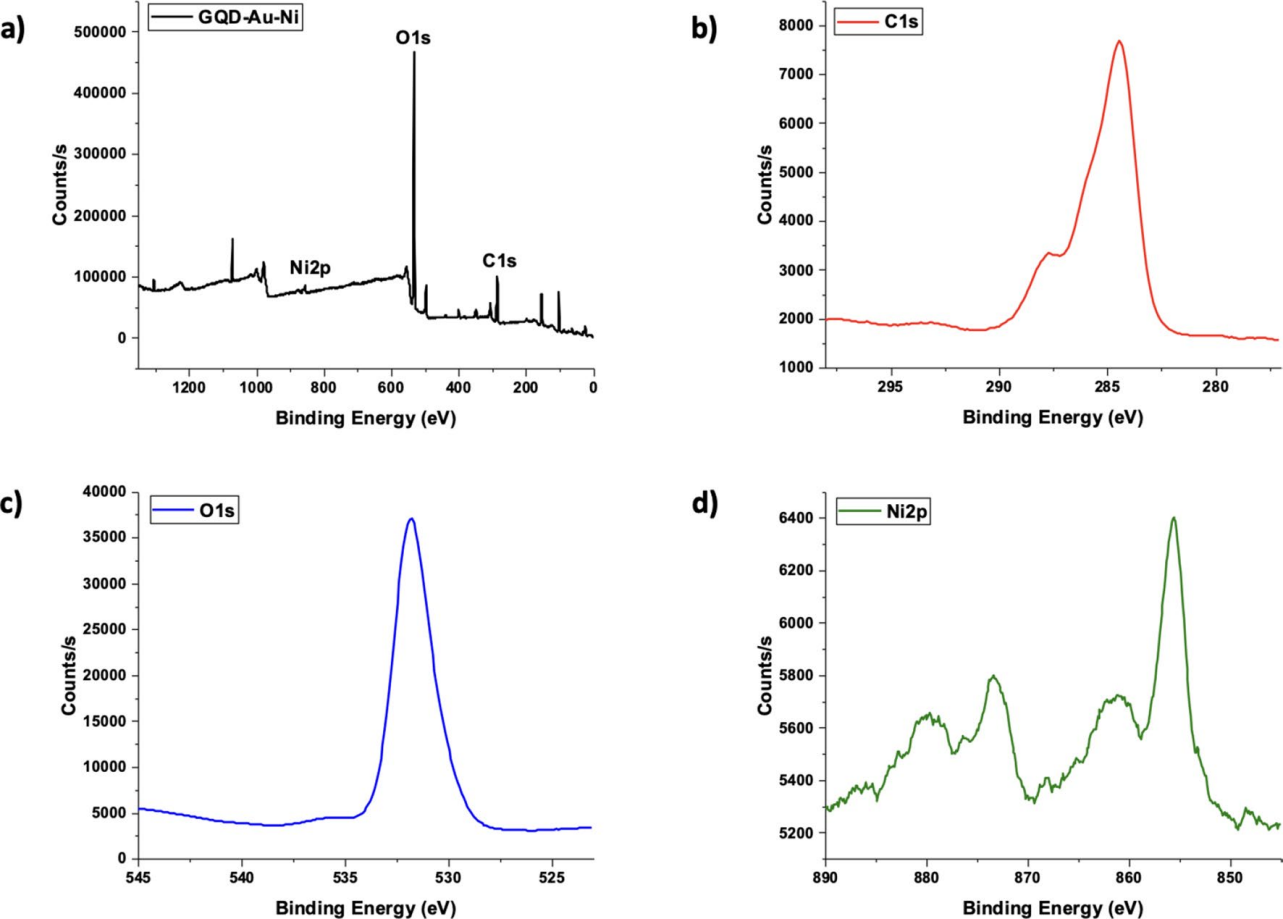
**a**) XPS spectrum of the GQD-Au-Ni micromotor **b**) C1s **c**) O1s **d**) Ni2p

The X-ray Photoelectron Spectroscopy (XPS) analysis graph of the GQD-Au-Ni nanomotor is presented in Fig. 4. The rising peak in the spectra shown in Fig. 4a and b can be attributed to C1s. This peak, representing the C-C/C-H bonds present in the GQD structure, was detected at a value of 284.54 eV [24–26]. Additionally, the rising peak at 287.79 eV can be attributed to the C = O bond [4]. The rising peak at a value of 531.93 eV in the spectra shown in Figs. 1c and 4a can be attributed to O1s [24, 27, 28]. In Fig. 4a and d, the two main characteristic peaks at 876.14 eV and 855.54 eV, originating from the Ni layer of the nanomotor, can be assigned to the Ni 2p₁/₂ and Ni 2p₃/₂ spin-orbit peaks [29]. These two characteristic peaks are followed by satellite peaks at 861.24 eV and 880.01 eV [30].

**Fig. 5:**
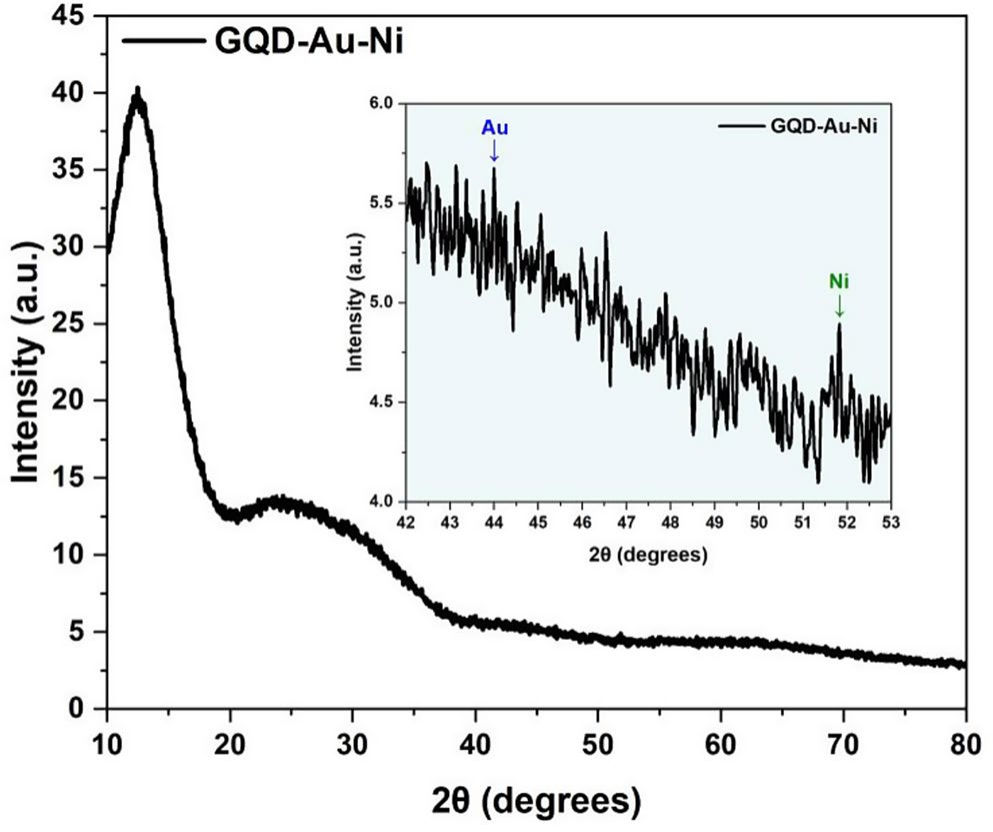
XRD patterns of GQD-Au-Ni micromotor

The results of the X-Ray Diffraction (XRD) spectroscopy analysis performed in the 2θ = 10° to 2θ = 90° range for the synthesized GQD-Au-Ni nanotube structure are presented in Fig. 5. Examination of the provided graph suggests that the synthesized structure is a product with weak crystallization [31]. A characteristic peak associated with GQD appears at 2θ = 24.35° [31, 32]. This broad peak, known to correspond to the (0.02) plane of GQD, can be attributed to the disordered stacking of GQDs [33]. Simultaneously, the sharp peak observed at 2θ = 24.35° corresponds to the diffraction peak of the GO (001) crystal face [34]. However, since the analysis was initiated at 2θ = 10°, it is not possible to comment on the presence of the diffraction peak of the (001) crystal face of GQD. In the GQD-Au-Ni tubular motor, a characteristic Au peak with a rather low peak intensity, masked by the GQD layer in the upper layer, is observed at 2θ = 43.9° [24]. Additionally, a minimal peak corresponding to the Ni atom appears at 2θ = 51.8° [24]. The masking of the characteristic Au and Ni peaks at 2θ = 43.9° and 2θ = 51.8°, respectively, is due to the weak crystallization tendency of the GQD structure [31].

SI Fig. 2 illustrates the EIS results for the bare Screen printed carbon electrode and the GQD-Au-Ni/SPCE electrode after interaction with 1 × 10^⁻5^ M Fe^3+^. EIS is an effective method for analyzing the surface characteristics and impedance of modified electrodes. In this method, the Rct value, which represents the electron transfer impedance parameter, is indicated by the diameter of the semicircle in the Nyquist plot [23]. The EIS graph in SI Fig. 2 consists of two regions: a semicircle in the high-frequency region and a linear section in the low-frequency region, attributed to diffusion control. The diameter of the semicircle corresponds to the Rct value on the electrode surface [23, 35, 36]. For materials with rapid charge transfer kinetics, the semicircle’s diameter is relatively small, whereas slower charge transfer results in a larger diameter. The Rct value can be determined as the diameter of the first semicircle in the Nyquist plot, and its magnitude is calculated using the equivalent circuit model [35]. Accordingly, the Rct value for the Bare electrode was calculated as 503.43 Ω, while the Rct value measured for the GQD-Au-Ni modified SPE surface was 521.91 Ω. The Rct of the bare SPE was quite small, but it decreased when GQD-Au-Ni micromotors were applied to the SPE surface, due to the increased conductivity of the GQD-Au-Ni micromotors. The Rct of the bare Screen-printed carbon electrode was quite small, but it decreased when GQD-Au-Ni micromotors were applied to the Screen-printed carbon electrode surface, due to the increased conductivity of the GQD-Au-Ni micromotors.

To optimize the incubation time of micromotors with Fe^3+^, incubation was performed with the highest iron concentration of 10^− 5^ M for different durations (5–120 min). As a result of the incubation, the speed of the micromotor was measured under an optical microscope. The micromotor, which lost about 10% of its speed in 5 min, decreased to approximately 0.5 μm/s after 60 min, and its speed remained almost the same at 120 min. As a result of the incubation, the optimal incubation time was selected as 60 min. The speed results of optimized incubation time are shown in SI Fig. 3.

**Fig. 6:**
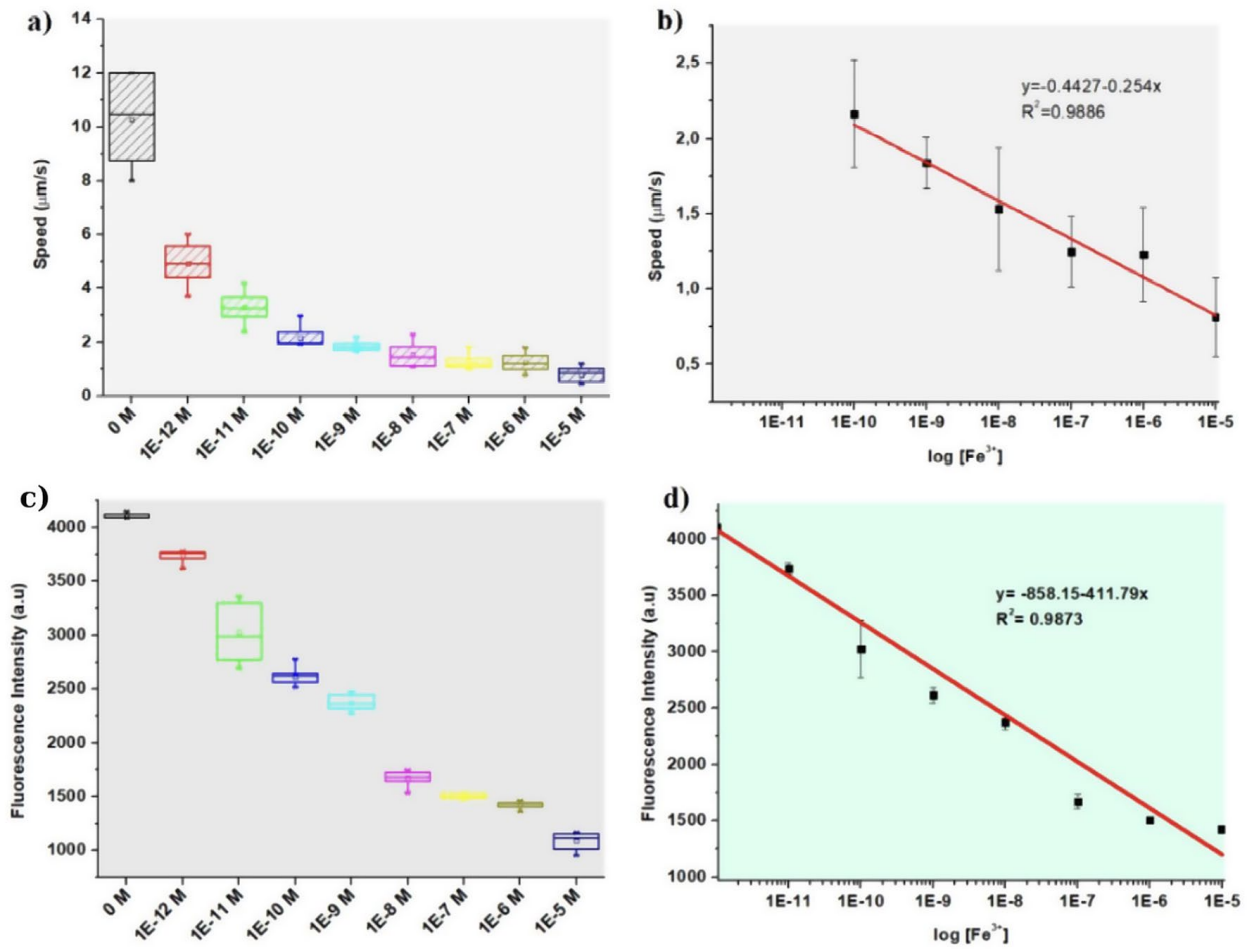
Effect of different concentrations of ferric ions on the **a**) speed and **b**) calibration curve based on speed of GQD-Au-Ni micromotors before and after Ferric ion incubation (*n* = 10). **c**) Effect of different concentrations of ferric ions on the fluorescence intensity **d**) Calibration curves based on fluorescence intensity (*n* = 10)

After different concentration of ferric ions with GQD based tubular micromotors for an hour, fluorescence intensity of the micromotors was measured by fluorescence microscopy. The presence of ferric ions also leads to dynamic fluorescence quenching in GQDs. This fluorescent quenching might result from changes in the local environment around the GQDs due to the interaction with ferric ions, leading to a decreased in fluorescence intensity [12, 37–39]. So, with increasing ferric ion concentration, micromotor fluorescence intensity decreased shown in Fig. 6a. Also because of the incubation of ferric ions speed of micromotor decreased with increased ion concentration was shown in Fig. 6c. Limit of detection was calculated according to the changes in the fluorescence intensity as well as the changes in the speed. As seen from the calibration curves in Fig. 6b and d, linearity was observed between increasing concentration and both fluorescence intensity and speed. LOD was found about 9 µM and 7 µM due to speed and fluorescence intensity respectively. Also, optical microscopy images of different ferric ion incubated GQD-Au-Ni micromotor shown in SI Fig. 3. Furthermore, One-way ANOVA test showed statistically significant differences between tested groups (different ferric ion concentration (*n* = 10)) by using Origin Pro 8.5. Regarding inter-group comparisons using Tukey’s test, there was a significant statistical difference between groups (*p*<0.05) were shown in Fig. 6a and c.

**Fig. 7:**
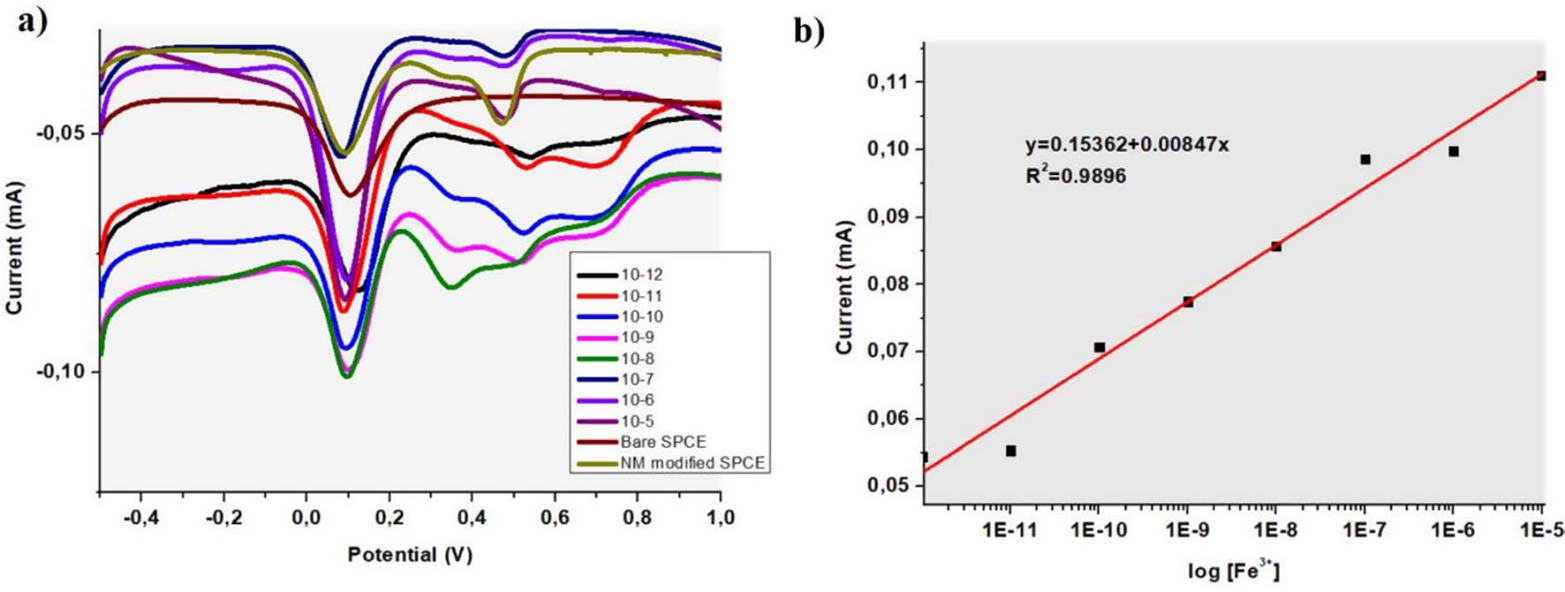
**a**) DPV curves of GQD-Au-Ni micromotors incubated with different ferric ion concentrations **b**) Calibration curve of DPV analysis

Further investigation for GQD-based micromotors additionally as an electrochemical DPV method for ferric ion sensing. According to DPV curves significant difference was observed between bare electrode (dark red line) and motor modified electrode (dark green line) (Fig. 7a). The effect of Fe³⁺ concentration on the electrochemical signal of the GQD-Au-Ni micromotors was investigated using various Fe³⁺ concentrations (from 1 × 10⁻¹² M to 1 × 10⁻⁵ M) as depicted in Fig. 7a. As the concentration of Fe³⁺ ions increased, the electrochemical response of the GQD-Au-Ni micromotors also increased. This is due to the alteration in electron density on the micromotor surface caused by the coordination of Fe³⁺ with the GQD-Au-Ni micromotors [40]. The observed peaks demonstrated a strong linear relationship between 1 × 10⁻¹² M and 1 × 10⁻⁵ M Fe^3+^ concentrations, as shown in Fig. 7b. From the linear curves, calculated LOD was about 6 µM.

**Fig. 8:**
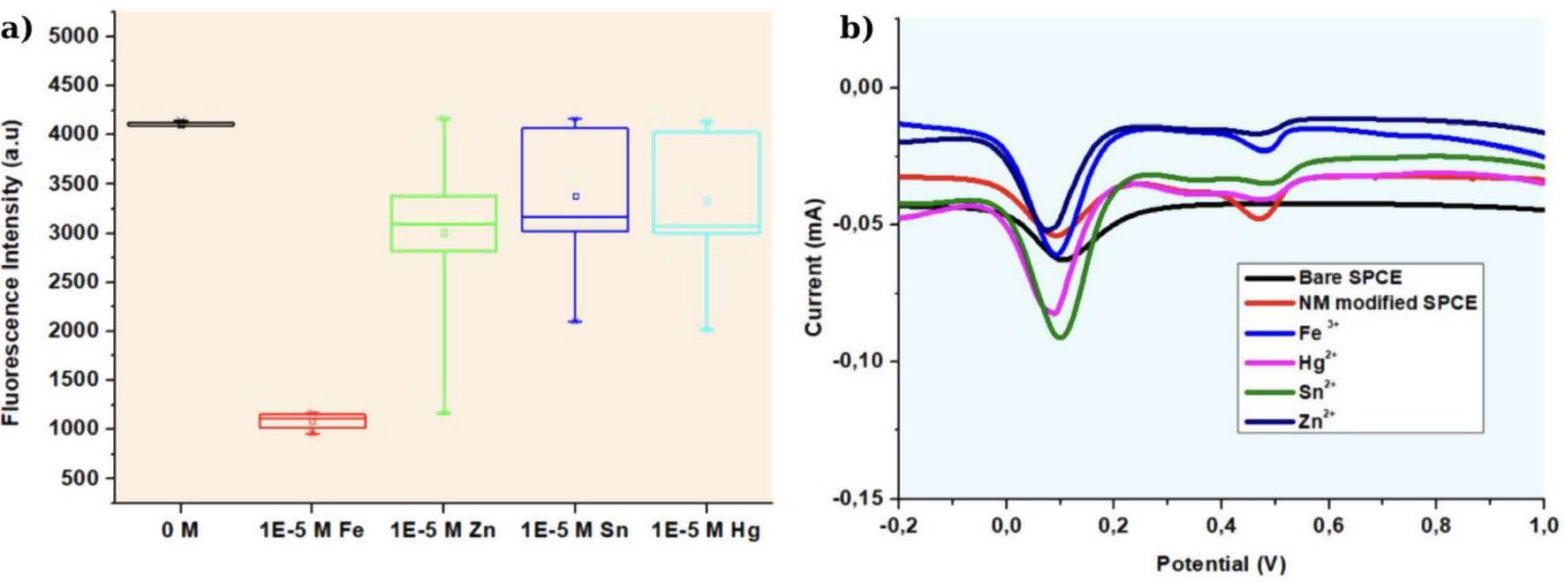
**a**) Fluorescence intensity of GQD-Au-Ni micromotors after incubated different metals **b**) DPV curves of GQD-Au-Ni micromotors after incubated different metals

The sensitivity of fluorescent-based tubular micromotors to different metal ions was investigated by measure the fluorescent activities followed both directly and in the presence of aqueous solutions of different metal ions. According to earlier research [41–45], the quenching of GQD photoluminescence can be attributed to the specific interaction between Fe³⁺ ions and oxygen/nitrogen functional groups, such as amino, carboxyl, or hydroxyl groups on the GQD surface. Due to varying bond lengths, the stability constants of metal ion complexes follow this order: Fe³⁺ > Al³⁺ > Cu²⁺ > Ni²⁺ > Pb²⁺ > Cd²⁺ > Co²⁺ > Ca²⁺ > Mg²⁺ > Ag⁺. Additionally, the paramagnetic nature of Fe³⁺ gives it a strong binding affinity for the electron-rich–OH or–NH₂ groups on the GQD surface [39] Fig. 8 shows that the results are consistent with the literature. In Fig. 8a, it is observed that after incubation with ferric ions at the highest concentration, the fluorescence intensity of the motor decreases, and there is also a decrease after incubation with Zn^2+^, Sn^2+^, and Hg^2+^, but the fluorescence intensity does not decrease as much as with iron. In Fig. 8b, selectivity for iron ions is confirmed by performing DPV electrochemical measurements for selectivity [40].

In summary, novel multifunctional GQD-Au-Ni micromotors were developed, which exhibited sensitivity to Fe^3+^ ions as evidenced by changes in fluorescence intensity, speed, and electrochemical response. Table 1 provides a comparison of materials selective for heavy metal ions as reported in the literature.

**Table 1:** Comparisons of metal ion determination with various sensor

Sensor	Ion	LOD	Reference
GQD	Fe^3+^	7.22 µM	[46]
DOPA-mediated fluorescent gold nanoclusters	Fe^3+^	3.5 µM	[47]
N-GQDs	Fe^3+^	0.87 µM	[39]
Nitrogen and Zinc doped carbon dots (N, Zn-CDs)	Fe^3+^	0.027 µM	[48]
Rhodamine based carbazole-Au-Ni micromotor	Hg^2+^	100 nM	[40]
Ag-Ni nanomotor	Ag^2+^	0.5–100 µM	[7]
GQD-Au-Ni	Fe^3+^	6 µM	This Study

**Fig. 9:**
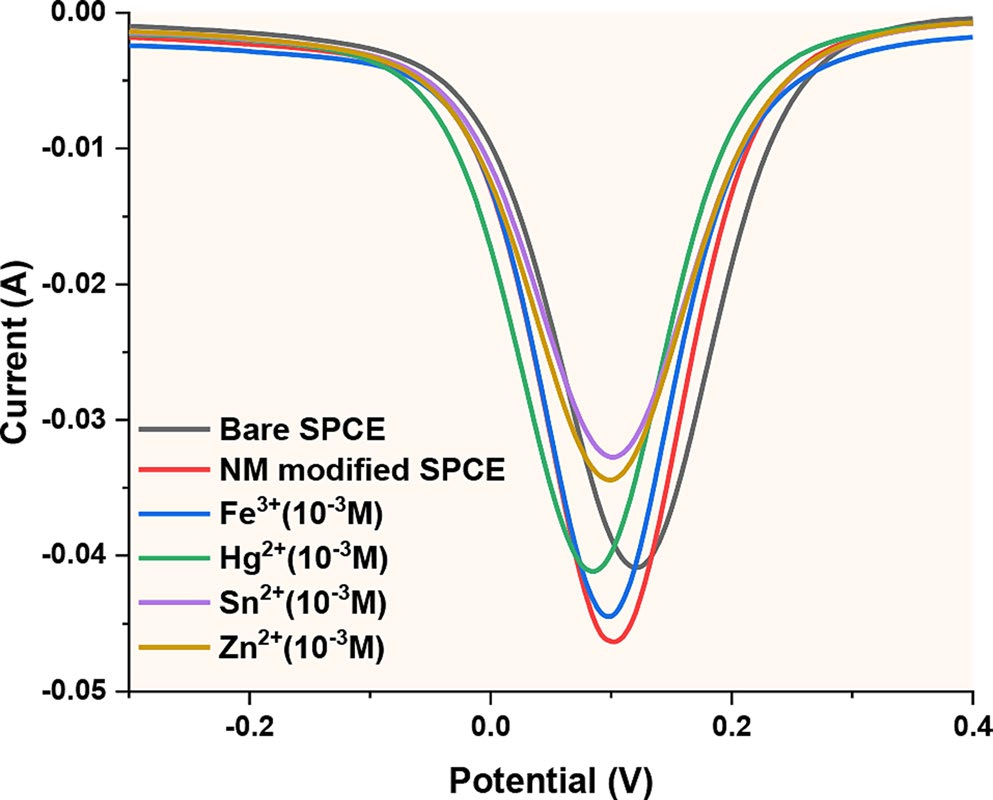
DPV curves of GQD-Au-Ni micromotors after incubated different metals with 10 times higher concentration

The GQD-Au-Ni nanomotor exhibits a characteristic oxidation peak at an appropriate potential in a 5 mM 5 mM Fe(CN)_6_^3−/4−^ electrolyte solution prepared in 0.1 M KCl. To detect Fe³⁺ ions, DPV analysis was performed at the determined potential, and interfering metal ions such as Hg²⁺, Sn²⁺, and Zn²⁺ were added at 10 times their concentration. The current changes were recorded, and the electrochemical response of the nanomotor to various metal ions is presented in Fig. 9. The electrochemical response is directly related to the heavy metal content of the nanomotor. As a result, the nanomotor containing Fe³⁺ ions exhibit a higher current density compared to other metal ions [49, 50]. In the presence of 100-fold interfering ions, the electrochemical response reductions of the nanomotor were recorded as follows: 3.98% for Fe³⁺ ions, 12.73% for Hg²⁺ ions, 29.47% for Sn²⁺ ions, and 25.48% for Zn²⁺ ions. Based on the observed electrochemical response results in the presence of 100-fold interfering ions, the GQD-Au-Ni nanomotor exhibits a selectivity performance of 96.02% towards Fe³⁺ ions.

## Conclusion

In this study novel graphene quantum dot-based gold-nickel (GQD-Au-Ni) micromotors successfully developed and characterized, which demonstrate exceptional sensitivity and selectivity for detecting ferric ions (Fe³⁺). These micromotors represent the first tubular GQD-Au-Ni micromotors reported in the literature, showcasing a significant advancement in micromotor technology. The integration of graphene quantum dots with gold and nickel layers not only enhances the micromotors’ fluorescence and magnetic properties but also facilitates rapid electron transfer, contributing to their high performance in sensing applications.

Our findings highlight the multifunctionality of these micromotors, employing fluorometric, magnetic, and amperometric methods to achieve selective and sensitive detection of Fe³⁺ ions. The micromotors exhibited a remarkable limit of detection (LOD) of 6 µM, outperforming many existing sensors. This sensitivity is crucial for monitoring ferric ions, which play a critical role in various biological and environmental processes, and can pose significant health and environmental risks at elevated levels.

The study provides a novel strategy for the development of advanced micromotors with potential applications in environmental monitoring and biomedical diagnostics. The promising results pave the way for future research into the development of multifunctional micromotors for a wide range of applications, including the detection of other heavy metal ions and pollutants. This work underscores the potential of GQD-based systems in revolutionizing sensor technology and addressing critical challenges in environmental and health monitoring.

## Electronic Supplementary Material

Below is the link to the electronic supplementary material.

Supplementary Material 1

Supplementary Material 2

## Data Availability

No datasets were generated or analysed during the current study.
