# Supplementary material for: Graphene Quantum Dot-Based Gold-Nickel Micromotors for Sensitive Detection of Ferric Ions
Source: J Fluoresc. 2025 Mar 18;35(10):9425–34. doi: 10.1007/s10895-025-04238-6 (PMC12672619; doi:10.1007/s10895-025-04238-6)
Supplement: Supplementary file 1 — Supplementary Material 1 [file 10895_2025_4238_MOESM1_ESM.docx]

**Development of Graphene Quantum Dot-Based Gold-Nickel Micromotors for Sensitive Detection of Ferric Ions**

Gozde Karaca^1^

^1^ Department of Medical Services and Techniques, Isparta Health Services Vocational School, Suleyman Demirel University 32260 Isparta, Turkey

**SI Figure Captions**

**SI Figure 1.** The mapping analysis of the GQD-Au-Ni micromotors a) GQD-Au-Ni micromotors b) Green İndicates Carbon element c) Blue indicates Oxygen elements d) Red indicates Nickel elements e) Yellow indicates Gold element

**SI Figure 2.** EIS results for the bare SPE electrode and the GQD-Au-Ni/SPCE electrode

**SI Figure 3.** Micromotor speeds after incubation 10^-5^ M Fe^3+^ with different durations

**SI Figure 4.** Optical microscopy images of different ferric ion incubated GQD-Au-Ni micromotor a) 10^-12^ M b) 10^-11^ M c) 10^-10^ M d) 10^-9^ M e) 10^-8^ M f) 10^-7^ M g) 10^-6^ M h) 10^-5^ M


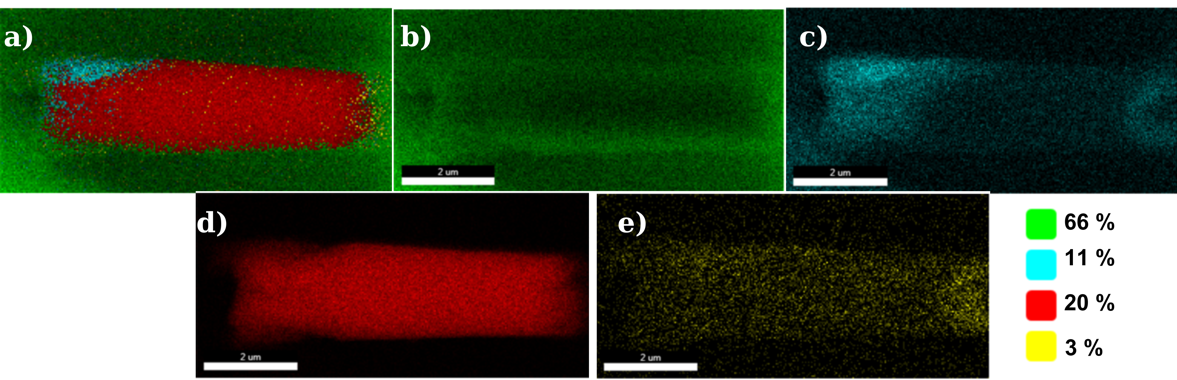


**SI Figure 1.** The mapping analysis of the GQD-Au-Ni micromotors a) GQD-Au-Ni micromotors b) Green İndicates Carbon element c) Blue indicates Oxygen elements d) Red indicates Nickel elements e) Yellow indicates Gold element


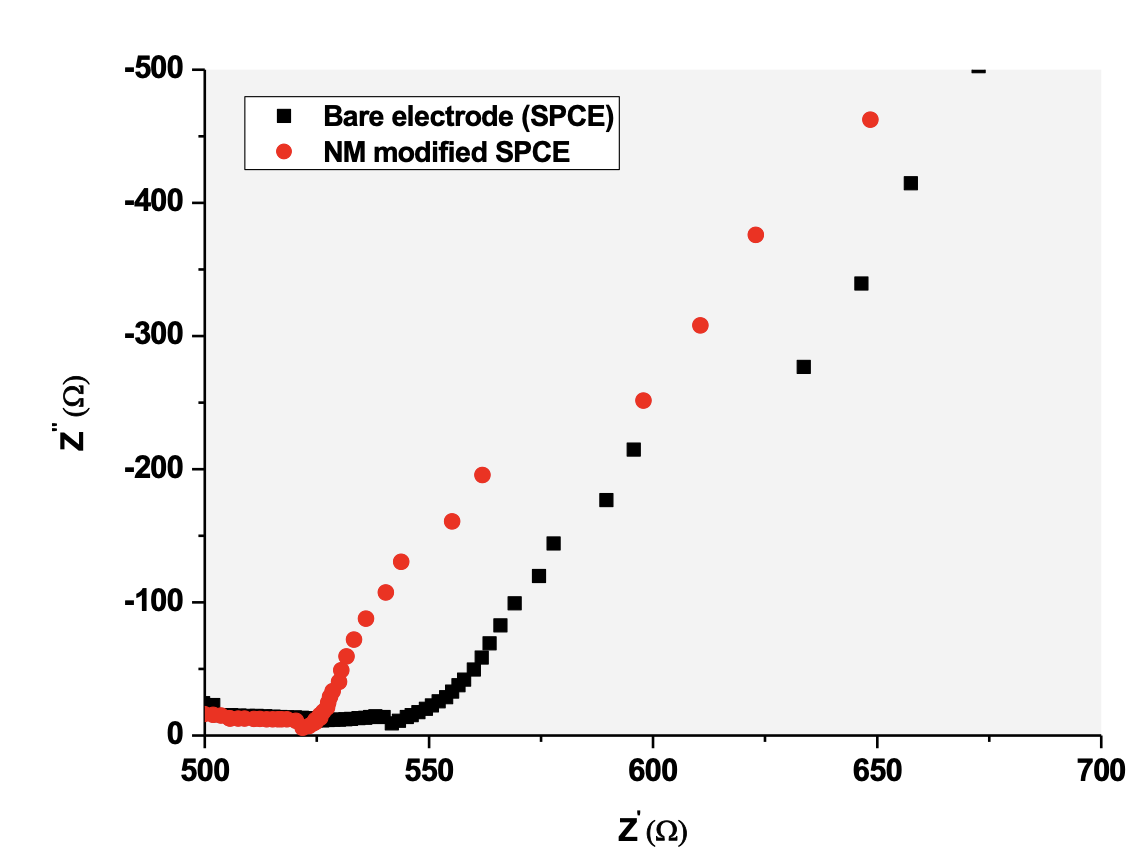


**SI Figure 2.** EIS results for the bare SPE electrode and the GQD-Au-Ni/SPCE electrode


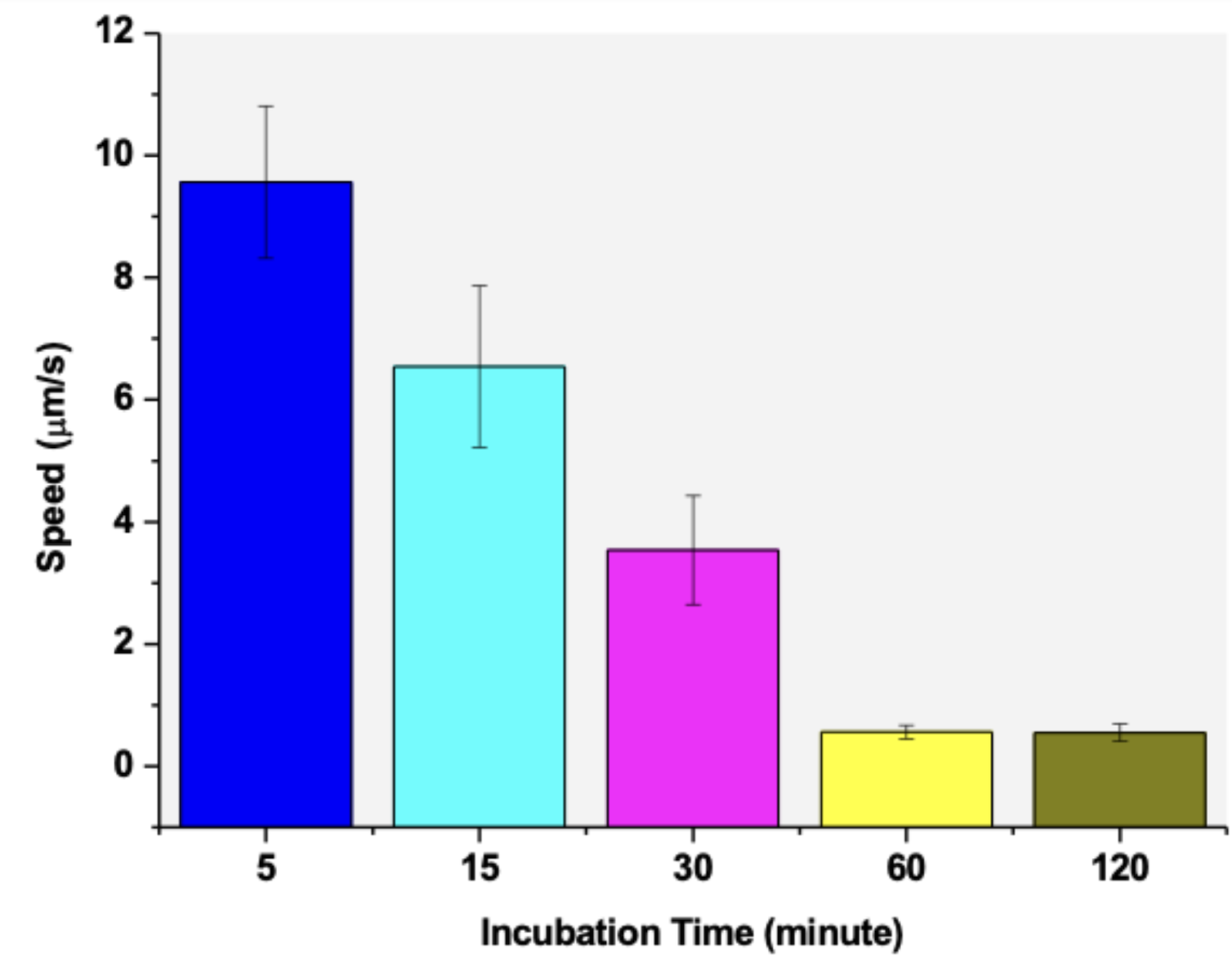


**SI Figure 3.** Micromotor speeds after incubation 10^-5^ M Fe^3+^ with different durations


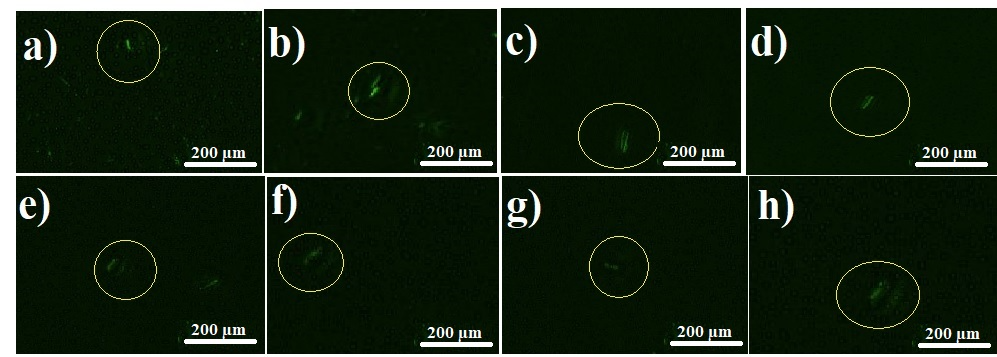


**SI Figure 4.** Optical microscopy images of different ferric ion incubated GQD-Au-Ni micromotor a) 10^-12^ M b) 10^-11^ M c) 10^-10^ M d) 10^-9^ M e) 10^-8^ M f) 10^-7^ M g) 10^-6^ M h) 10^-5^ M
